# Proposing new lipoprotein (a) cut off value for Kazakhstan: pilot study

**DOI:** 10.3389/fcvm.2024.1468566

**Published:** 2024-10-21

**Authors:** Makhabbat Bekbossynova, Marat Aripov, Tatyana Ivanova-Razumova, Aknur Kali, Dana Tleubayeva, Gulnur Daniyarova, Alexey Goncharov

**Affiliations:** ^1^ Heart Center, “University Medical Center” Corporate Fund, Astana, Kazakhstan; ^2^ Clinical Academic Department of Interventional Cardiology, Heart Center, “University Medical Center” Corporate Fund, Astana, Kazakhstan; ^3^ Pediatric Department, Mother and Child Center, “University Medical Center” Corporate Fund, Astana, Kazakhstan; ^4^ Department of Cardiology, Heart Center, “University Medical Center” Corporate Fund, Astana, Kazakhstan; ^5^ Research Department, “University Medical Center” Corporate Fund, Astana, Kazakhstan

**Keywords:** lipoprotein (a), low-density lipoprotein cholesterol, atherosclerotic cardiovascular diseases (ASCVD), aortic stenosis, ethnicity

## Abstract

**Introduction:**

There is no consensus on the optimal concentration of lipoprotein(a) (Lp(a)) for the risk of atherosclerotic cardiovascular diseases (ASCVD) and aortic valve stenosis. In various clinical guidelines and agreed documents, the threshold level of Lp (a) is 30 mg/dl or 50 mg/dl. We estimated the cut-off value of Lp (a) associated with the risk of developing various localizations of atherosclerosis for the Central Asia, including Kazakhstani population.

**Methods:**

This study was conducted at National Research Cardiac Surgery Center, Kazakhstan. 487 patients were included, of which 61.3% were men. The mean age of all participants was 57.3 ± 12.6 years. Bivariate and multivariable logistic regression analysis was used to study the relationship between risk factors and plasma lipoprotein (a) levels. The threshold value of lipoprotein (a) was predicted using the Youden index.

**Results:**

For Kazakhstani population the lipoprotein (a) cut offs for the risk of developing atherosclerotic CVD and aortic valve calcification was 21.1 mg/dl (*p* < 0.05). There was no relationship with the level of lipoprotein (a) and low-density lipoprotein cholesterol (LDL-C), which suggests that lipoprotein (a) is an independent risk factor for the development of ASCVD.

**Discussion:**

This study offers new insights into the threshold value of lipoprotein (a) in Kazakhstan, highlighting its role as a risk factor for atherosclerotic cardiovascular diseases and aortic valve calcification. The findings suggest that the internationally recommended Lp(a) cutoffs may not be suitable for Central Asian populations, as the threshold in our study is significantly lower at 21.2 mg/dL. These results emphasize the need for further research with larger sample sizes to establish more region-specific cutoffs.

## Introduction

The American College of Cardiology/American Heart Association (AHA/ACC) and European Society of Cardiology/European Atherosclerosis Society guidelines on cardiovascular risk unanimously confirm that elevated lipoprotein (Lp(a)) remains an independent risk factor for atherosclerotic cardiovascular disease (ASCVD) (1, 2).

High Lp(a) concentration is caused by genetic factors and the damaging effects are mediated by multiple mechanisms including proinflammatory, procalcifying and proatherogenic pathways, which are attributed to the oxidised phospholipid content of Lp(a) (3).

Meta-analysis of a total of 5,436 CHD patients from 27 prospective studies concluded that people with elevated Lp (a) levels are up to 70% higher risk of developing CHD than those with normal Lp (a) levels (4).

Worldwide, experts estimate the number of people with elevated Lp (a) levels to be more than 1 billion. Approximately 10% of the general Asian population may have Lp (a) > 50 mg/dl compared with 15%–30% of the world population (5).

However, currently there is no single clinical threshold for Lp(a) to differentiate increased risk of developing ASCVD. Clinical laboratories in the USA define an elevated Lp(a) level at 30 mg/dl, the American Heart Association/American College of Cardiology and European guidelines recommend a threshold of 50 mg/dl (1, 2), but the British Biobank study shows that cardiovascular risk begins at much lower levels (6).

The fact that Lp(a) may differentially increase the risk of atherosclerotic CVD outcomes according to race/ethnicity further confuses the issue (3–6). The mechanism underlying these differences is dominated by a genetic factor.

Numerous prospective studies have shown that black people have 2- to 3-fold higher levels of Lp(a) than white people. A small number of studies conducted in Chinese and Hispanics have found that Chinese have lower levels of Lp (a) than white people, whereas conflicting results have been obtained in Hispanics (7, 8).

In Central Asia, including Kazakhstan, the prevalence of ASCVD has almost doubled over the last 40 years and to present day there are no studies devoted to determining Lp(a) thresholds for this population.

In this regard, the aim of our study was to investigate the threshold value of Lp(a) in the adult population of Kazakhstan and to identify the relationship with the development of ASCVD.

## Materials and methods

The study is retrospective and single-center. All patients over 18 years of age, who were hospitalized at the National Research Cardiac Surgery Center and whose plasma Lp(a) level was determined, were included in the study within the period from January 2023 to September 2023. Thus, 487 patients with the required clinical, laboratory and instrumental data were included in the study.

Baseline clinical data including age, sex, body mass index, arterial hypertension, smoking, diabetes mellitus, coronary heart disease, aortic valve disease, peripheral arterial disease, lipid profile (including total cholesterol, LDL, HDL, triglycerides, Lp(a), apolipoprotein B levels were obtained from medical records.

Dependent variables included arterial hypertension, type 2 diabetes mellitus (T2DM), dyslipidemia, defined as low-density lipoprotein cholesterol >130 mg/dl, high-density lipoprotein cholesterol (HDL-C) < 45 mg/dl, triglycerides (TG) > 100 mg/dl; ischemic heart disease determined by coronary angiography, aortic valve calcification based on echocardiography.

## Statistical analysis

Statistical analyses were performed applying Stata (version 3.6.3). Data were presented as mean values ± standard deviation (SD) or medians for quantitative variables and percentages for qualitative variables. Differences in clinical and biochemical parameters between groups were evaluated using Student's *t*-test, Mann-Whitney *U*-test, Kraskell-Wallis Criterion, respectively. Patients all signed informed consent and the local ethical committee (approval number № 2023/01-008 from 05.07.2024) approved the study.

Signiﬁcance level for all statistical tests was set at 0.05. We used receiver-operator characteristics (ROC) curves to determine the optimal BMI or WC cut-points for identifying general or central obesity.

Signiﬁcance level for all statistical tests was set at 0.05. ROC curves were used to determine the optimal Lp(a) cut-offs based on the Youden index. A higher Youden index indicates greater accuracy. Since Youden's statistic has the lowestmean squared error (MSE) and bias, it was more appropriate for use.

## Results

At baseline, 487 patients’ data were used for analysis. 63.1% of them are men and the mean age of all participants was 57.3 ± 12.6 years. Table 1 shows the clinical and demographic characteristics of patients.

**Table 1:** Characteristics of the study population (total *n* – 487).

Variables	Low-Lp(a) < 30 mg/dl (*n* = 352)	High Lp(a) > 30 mg/dl (*n* = 135)	*p*
Age (years)	56.6 ± 12.6	59.7 ± 12.3	0.01
Male (*n* – 311)	222 (63.1%)	88 (65.2%)	0.7
Asian	301 (86%)	51 (37.7%)	0.1
European	107 (30.3)	27 (20%)	
BMI (kg/m^2^)	29.0 ± 5.0	28.9 ± 4.9	0.8
Arterial Hypertension	249 (70.4%)	106 (78.5%)	0.52
Smoking	84 (23.9%)	47 (34.8%)	0.07
Diabetes mellites type 2 (T2DM)	75 (21%)	22 (17%)	0.1
Ishemic heart disease (IHD)	127 (36.1%)	80 (59.2%)	<0.001
Aortic valve lesion	45 (12.7%)	30 (22.2%)	0.02
PDA (Doppler)	159 (45.1%)	86 (64.4%)	<0.01
Total Chol (mg/dl)	195.4 ± 60.4	192.8 ± 54.1	0.6
LDL (mg/dl)	120.2 (81.0–157.0)	122.2 (90.4–160.3)	0.5
HDL (mg/dl)	45.9 (36.3–57.0)	47.2 (39.9–55.9)	0.4
Trig (mg/dl)	89.4 (52.1–148.5)	92.3 (58.5–137.8)	0.8

According to European guidelines, all patients are divided into two categories: the high level of lipoprotein (a) ≥ 30 mg/dl and low lipoprotein (a) < 30 mg/dl. Of the 487 patients, 135 (27.7%) had high Lp(a) levels, and 352 (72.3%) had low Lp(a) levels. The group with normal Lp(a) levels had a higher prevalence of Asian adults (86%) compared to the group with high Lp(a) levels (37.7%) (*p* < 0.1). High Lp(a) levels were positively associated with aortic valve lesion (*p* = 0.02), peripheral artery disease (PAD) (*p* < 0.01), and ischemic heart disease (IHD) (Table 1).

**Figure 1:**
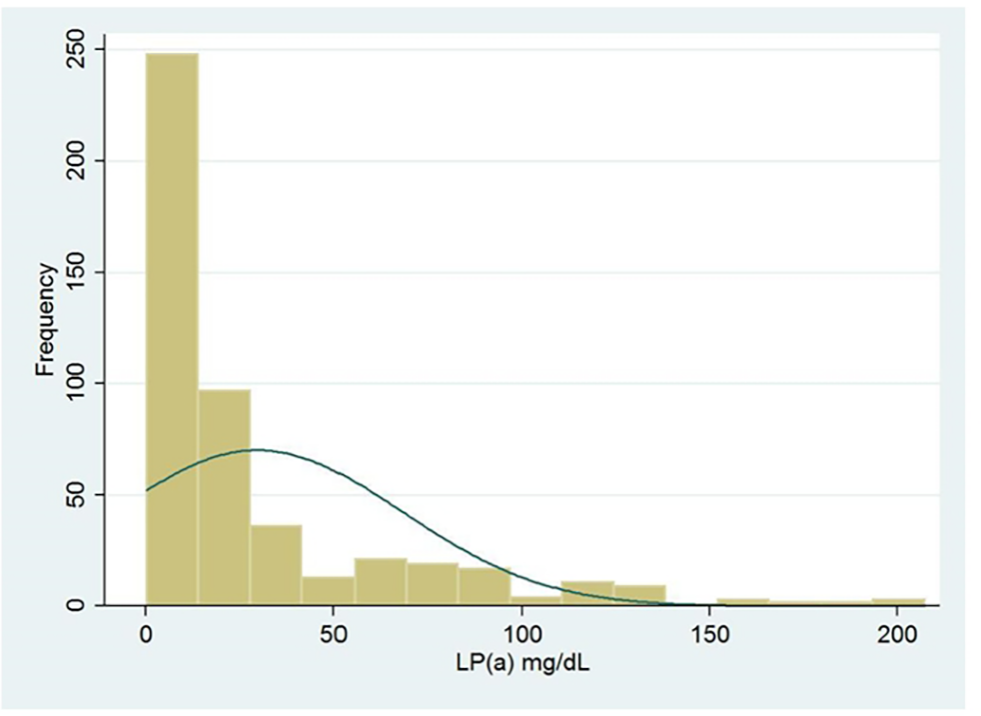
Histogram of lipoprotein (a) (Lp(a)) distribution frequency.

**Figure 2:**
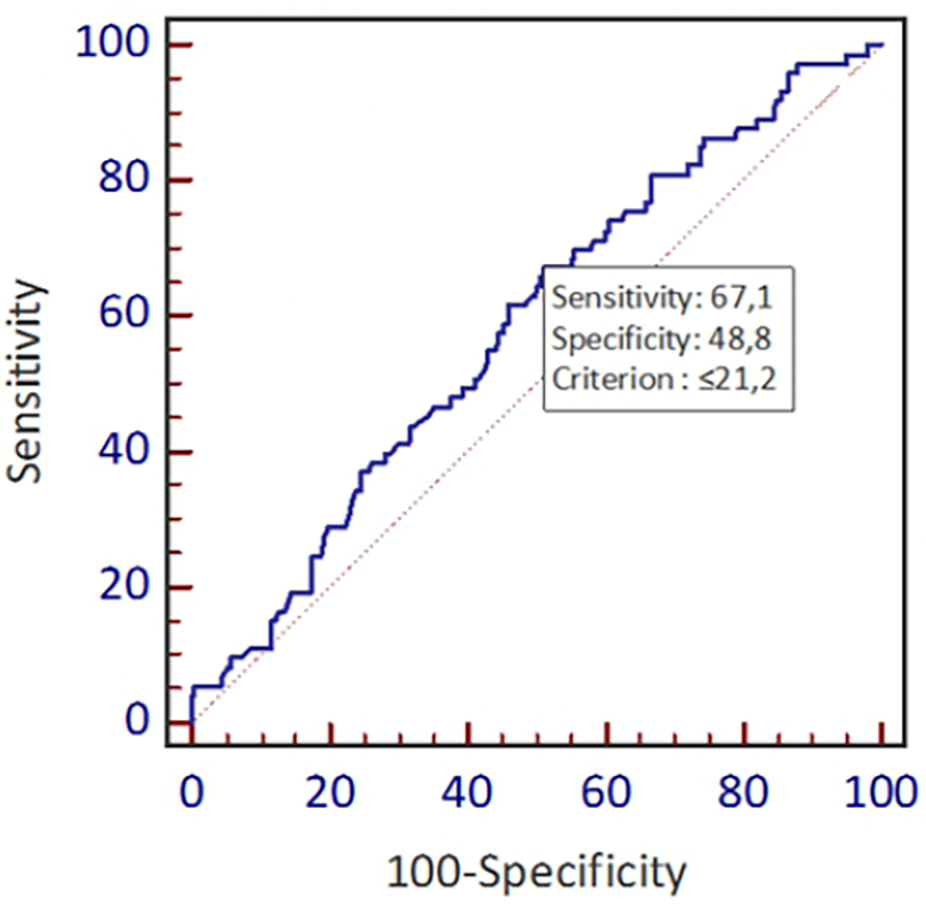
New threshold value for lipoprotein (a) and new cut-off.

Figure 1 shows the distribution frequency of lipoprotein (a). Lipoprotein (a) levels in our population had a skewed distribution with a tail toward the highest values with a mean of 42.2 mg/dl, which is consistent with earlier studies (Figure 1) (9). Since Asian population was considered to have lower cut-off values during literature review, we used the ROC curve with Youden index to predict a new threshold for lipoprotein (a) distinctively for our population (Figure 2). The cutoff value for Lp(a) for further analysis was 21.2 mg/dl, calculated using the ROC curve test associated with the Youden index (*J* = 0.2). At this level, sensitivity was 0.67 and specificity was 0.49 for ASCVD.

Table 2 shows the positive correlation between IHD, aortic valve stenosis and PDA with the new lipoprotein (a) cut offs (*p* < 0.01). There was no relationship with the level of lipoprotein (a) and low-density lipoprotein cholesterol (LDL-C), high-density cholesterol which confirmed that lipoprotein (a) is an independent risk factor for the development of ASCVD.

**Table 2:** New cut off for Lp (a) by ROC curve (*n* = 487).

Variables	Low-Lp(a) < 21.2 mg/dl (*n* = 312)	High Lp(a) > 21.2 mg/dl (*n* = 175)	*p*
Age (years)	56.4 ± 12.8	59.2 ± 12.0	0.02
Male (*n* – 311)	193 (61.8%)	117 (66.8%)	0.3
BMI (kg/m^2^)	28.9 ± 4.9	29.0 ± 5.1	0.8
Arterial Hypertension	219 (70.1%)	136 (77.7%)	0.8
Smoking	74 (23.7%)	57 (32.5%)	0.2
Diabetes mellites type 2 (T2DM)	63 (21%)	34 (17%)	0.6
Ischemic heart disease (IHD)	106 (33.9%)	101 (57.7%)	<0.001
Aortic valve lesion	39 (12.5%)	36 (20.5%)	0.04
PDA (Doppler)	140 (45.0%)	105 (64.4%)	<0.01
Total Chol (mg/dl)	196.7 ± 59.9	191.1 ± 56.4	0.3
LDL (mg/dl)	120.9 (81.7–157.3)	118.5 (83.4–158.9)	0.9
HDL (mg/dl)	46.0 (36.3–57.9)	45.9 (38.5–55.7)	0.7
Trig (mg/dl)	93.9 (54.5–148.9)	89.5 (52.1–136.2)	0.4

## Discussion

High levels of Lp (a) increase the risk of premature development of CHD, ischemic stroke, peripheral arterial disease (PAD) and aortic valve calcification (10, 11). Using UK Biobank EAS data the 2022 consensus conspiracy showed that compared to people with an average Lp(a) concentration of 7 mg/dl, people with levels of 30, 50, 75, 100 and 150 mg/dl had an increased risk of atherosclerotic CVD of 1.22, 1.40, 1.65, 1.95 and 2.72 times, respectively (12).

Nevertheless, the threshold value of Lp(a) is still an open question and this may affect the stratification of cardiovascular risk category. In this regard, the EAS 2022 consensus statement suggested a pragmatic approach: for “exclusion” <30 mg/dl, for “inclusion” >50 mg/dl, also an intermediate gray zone of 30–50 mg/dl.

In the study by Yoshiyasu Minami et al. the threshold value of Lp(a), which has become a risk factor for IHD in the West Asian population is 30 mg/dl (13), but Kim et al. suggest that for East Asians this value is equal to 26.3 mg/dl (14). Nevertheless, to this day, no data exist for Central Asia. Thus, in our study we determined the threshold value of Lp (a) for the population of Kazakhstan, which was 21.2 mg/dl (*p* < 0.05), where this parameter is lower than in East Asia.

The mechanisms by which Lp(a) contributes to the development of aortic valve calcification is a procedure, similar to coronary artery atherosclerosis. Inflammation, lipid deposition, fibrosis and calcification constitute the histological features observed in both conditions. The content of oxidized phospholipids (OxPL) in apolipoprotein (a) and its proinflammatory properties are one of the key factors in aortic valve stenosis. It is believed that inflammation leads to the activation of the calcification process by activating the innate immune response (15). Zheng et al. found that high levels of lp(a) and OxPL-apoB were independently associated with increased active tissue calcification and clinical phenomena such as aortic valve calcification (13). Taking into account the similar mechanisms of development, these pathologies were included in our research (16).

In most studies, the incidence of aortic valve stenosis was higher in the high Lp(a) group than in the low Lp(a) group (17). The pooled results of the lp(a) 50 mg/dl group confirmed that plasma lp(a) levels ≥50 mg/dl may be a risk factor for aortic valve lesions and there was insufficient evidence of an association between Lp(a) levels ≥30 mg/dl in plasma. In addition, the significant heterogeneity between studies was not well explained by subgroup analysis with an Lp(a) threshold of 30 mg/dl, requiring caution in interpreting the results (3). Patients with plasma Lp(a) levels ≥50 mg/dl may have a higher risk of than patients with lp(a) ≥ 30 mg/dl.

In our study, we also evaluated the relationship between elevated plasma Lp(a) levels and the risk of aortic valve lesions. The incidence of atherosclerotic cardiovascular disease (CVD) 59.2% was higher in the group with high Lp(a) levels >30 mg/dl than in the group with low levels, a finding supported by Gissette Reyes-Soffer et al. (18).

The question of whether lipoprotein (a) is an independent risk factor for atherosclerotic IHD remains relevant. Moreover, in this study, there was no correlation between LDL-C and plasma Lp (a) levels, indicating that lipoprotein (a) may be an independent risk factor for IHD and aortic valve stenosis. Some studies have reported that the risk of IHD due to elevated Lp (a) is exacerbated in the presence of other lipid risk factors, such as high LDL cholesterol or low HDL cholesterol (9, 19, 20).

The question of changes in plasma lipoprotein (a) levels throughout life remains an important issue. In this study, there was no relationship between age, sex and Lp (a) levels. However, in the study of Sofie Bay Simony et al. lipoprotein (a) concentrations increased moderately with age, especially in women, but the risk of morbidity and mortality due to high lipoprotein(a) levels was similar in women and men over 50 years of age. This means that elevated lipoprotein(a) levels over 50 years of age are a relatively more common cardiovascular risk factor in women, indicating the need for repeated measurements in women over 50 years of age (15).

Thus, this study is the first in Central Asia where the threshold level of lipoprotein (a) was determined.

There were some limitations to this study as this was a pilot study using a small sample. First, sample size was small (*n* = 487) to perform further stratification analyses. Additionally, study used data only from National Research Cardiac Surgery Center, it is imperative to note that findings should not be generalized to the entire Kazakhstani population. As a result, larger studies are required to back-up our findings and further it is planning to do as part of this research.

## Conclusion and recommendations

Research has shown the clinical and practical importance of determining the level of Lp (a). These results suggest that the threshold value for the population of Central Asia, in particular Kazakhstan, is 21.2 mg/dl, which is significantly lower than the generally accepted values. This finding emphasizes our hypothesis that there are different thresholds among different ethnic groups and requires further investigation.

## Data Availability

The raw data supporting the conclusions of this article will be made available by the authors, without undue reservation.
